# CoR-MAC: Contention over Reservation MAC Protocol for Time-Critical Services in Wireless Body Area Sensor Networks

**DOI:** 10.3390/s16050656

**Published:** 2016-05-09

**Authors:** Jeongseok Yu, Laihyuk Park, Junho Park, Sungrae Cho, Changsup Keum

**Affiliations:** 1 School of Computer Science and Engineering, Chung-Ang University, 221 Heukseok, Dongjak, Seoul 156-756, Korea; jsyu@uclab.re.kr (J.Y.); lhpark@uclab.re.kr (L.P.); jhpark@uclab.re.kr (J.P.); 2 Electronics and Telecommunications Research Institute, 218 Gajeong-ro, Yuseong-gu, Daejeon 34129, Korea

**Keywords:** wireless body area sensor networks, urgent data, priority level

## Abstract

Reserving time slots for urgent data, such as life-critical information, seems to be very attractive to guarantee their deadline requirements in wireless body area sensor networks (WBASNs). On the other hand, this reservation imposes a negative impact on performance for the utilization of a channel. This paper proposes a new channel access scheme referred to as the contention over reservation MAC (CoR-MAC) protocol for time-critical services in wireless body area sensor networks. CoR-MAC uses the dual reservation; if the reserved time slots are known to be vacant, other nodes can access the time slots by contention-based reservation to maximize the utilization of a channel and decrease the delay of the data. To measure the effectiveness of the proposed scheme against IEEE 802.15.4 and IEEE 802.15.6, we evaluated their performances with various performance indexes. The CoR-MAC showed 50% to 850% performance improvement in terms of the delay of urgent and time-critical data according to the number of nodes.

## 1. Introduction

Spurred by the rapid convergence of key technologies, such as physiological sensors, wireless communication and low power micro-electro-mechanical systems (MEMS), a new generation of wireless body area sensor networks (WBASNs) is emerging. These WBASNs exploit special-purpose implantable biosensors that aim to provide a large variety of applications, including computer-assisted rehabilitation, early detection of medical conditions, *etc*. To display human physiological status, these biosensors continuously monitor and transmit health conditions to a coordinating device wirelessly, forming a WBASN.

Although the applications of these WBASNs are attractive, there are still a large number of challenges to be tackled for providing reliable and efficient WBASN systems. One of the important challenges is to provide urgent data transmissions for life-critical applications. Techniques for urgent data transmissions have traditionally been based on *priority*. There have been a large number of studies on priority-based mechanisms [1,2,3,4,5,6] in wireless networks. However, these mechanisms are not applicable to WBASNs, since they are not designed for a WBASN’s unique features of low-power, reliability and the accommodation of diverse traffic types, including emergency alarms.

In addition to the above schemes, a number of studies related to WBASN are based on the IEEE 802.15.4 technology [7,8,9,10,11]. The IEEE 802.15.4 has a superframe structure consisting of a contention-free phase (CFP) and a contention access phase (CAP). If we use the CAP to transmit urgent data, there is no guaranteed deadline of urgent data transmission due to contentions with other urgent or non-urgent data. (the IEEE 802.15.6 recommends 20 ms for this deadline [12]). Therefore, to guarantee such a deadline, the CFP is a better option for urgent data transmission. However, the IEEE 802.15.4 standard has only seven guaranteed time slots (GTSs) in the CFP, which is likely not sufficient to accommodate additional devices. Further, since the emergency alarms do not occur frequently, reserving the GTSs for emergencies will waste scarce wireless resources.

For WBASNs, the few proposals for urgent data transmission in terms of priority-based MAC can be classified into three categories: *parametric* [13,14,15], *channelization-based* [16,17,18,19] and *hybrid* [20,21] approaches. The parametric approach is a technique where channel access parameters are treated differently for various traffic types, as in the enhanced distributed channel access (EDCA) mechanism in IEEE 802.11e [22]. The channelization-based approach is a scheme in which priority is assigned based on transmission opportunity. For instance, GTSs can be assigned to urgent traffic without contention, while non-urgent traffic is transmitted over the CAP with more contentions, as in the IEEE 802.15.4. The hybrid approach is a scheme that exploits the benefits of both parametric and channelization-based techniques. Recently, IEEE 802.15.6 produced a new WBASN standard that can be considered a hybrid approach. Details of this classification will be discussed in Section 2.

In this paper, we propose a hybrid priority-based MAC protocol for WBASN, referred to as contention over reservation MAC (CoR-MAC). In the CoR-MAC, each time slot is dedicated to a node that might transmit urgent data. However, dedicated time slots waste wireless resources. When the dedicated time slots are not used, the other nodes cannot use those time slots. To solve this problem, in the CoR-MAC, data can be transmitted over the time slot, which is dedicated to another node and not in use.

The rest of this paper is organized as follows. We survey the existing related work in Section 2. In Section 3, we describe the proposed CoR-MAC in detail, followed by the theoretical analysis of delay in Section 4. Section 5 evaluates the performance of our protocol compared to the existing schemes. Finally, we draw conclusions and suggest future directions in Section 6.

## 2. Related Work

According to the taxonomy mentioned in Section 1, Cao *et al.* [13] proposed a parametric prioritization scheme for QoS provisioning in WBASNs. In this protocol, WBASN traffic is divided into three classes of alarm/control (AC), command/data (CD) and routine (RT) traffic in a descending order of priority (*i.e.*, the AC traffic has the highest priority). To differentiate AC and CD traffic, a unique backoff exponent (BE) is configured for each. In other words, the AC has more transmission to opportunities than the CD, since the AC has a smaller BE. Nevertheless, there is no way that the AC can preempt the CD when the CD has already decreased its backoff counter to be smaller than that of the AC. Zhang *et al.* [14] proposed another parametric prioritization protocol in which traffic is divided into two classes of life-critical medical and other. To guarantee the QoS of the life-critical traffic, they exploit two CAPs in the protocol. Their CAP is further divided into two parts: AC1 for the life-critical medical uplink control and AC2 for the other uplink control. During AC1 and AC2, time slots are reserved for periodic traffic and bursty traffic. Although divided time slots can help to transmit bursty traffic with heavy-weighted periodic traffic, the time slots may cause negative impact on the utilization of wireless resources because bursty traffic and periodic traffic neither use timeslots of the other side. Ali *et al.* [15] suggested different maximum retry limits for transmission of emergency data and normal data in order to provide a higher probability of emergency data transmission. However, this scheme only provides reliable emergency data transmission, but does not guarantee the delay of urgent data.

Zhang *et al.* [16] proposed a priority-guaranteed MAC protocol as a channelization-based approach. In this protocol, data traffic is divided into two classes of medical (urgent) and consumer electronic (CE), both of which are transmitted over TDMA slots. To reserve their TDMA slots, both traffic types use a CAP. Since the CAP and CFP are divided into two, respectively, for both types of traffic, the medical traffic can still access the channel even if the CE traffic is overwhelmed. This is their prioritization mechanism differentiating medical from CE traffic. However, their approach does not guarantee timely transmission of urgent medical data, even if TDMA slots for medical traffic are exhausted, but the slots for CE are available. Li *et al.* [18] proposed an IEEE 802.15.4-based MAC protocol that adopts the slotted Aloha mechanism for the reservation of a GTS or normal data transmission in the CAP with a mini-slot. If non-urgent data win a contention in CAP, urgent data cannot be transmitted in the superframe, and it does not guarantee the QoS. Otal *et al.* [17] proposed the distributed queuing body area network (DQBAN) targeting both the QoS of urgency data and energy efficiency. The DQBAN utilizes its cross-layer fuzzy rule-based scheduler to schedule higher-priority urgent data before the other traffic types in the data transmission queue (DTQ). However, there still remains the problem of the channelization-based protocol, which cannot guarantee the deadline of urgent data transmission due to contentions with other data. Zhen *et al.* [19] proposed a channelization-based MAC protocol based on IEEE 802.15.4. In [19], the priority access period (PAP) is added after the beacon phase in the superframe for urgent data transmission. While the proposed scheme targeted reducing the delay of urgent data, PAP may reduce the throughput since the PAP phase is used only for urgent data. However, the channelization-based technique has the problem of bandwidth wastage. In other words, if excessive time slots for urgent data are reserved, bandwidth is wasted. This also imposes a negative impact on throughput performance for non-urgent data. In contrast, if minimal time slots are allocated for the urgent data, timely transmission of these data might not be possible. Therefore, the optimum number of time slots for urgent data should be computed for the channelization-based technique, which is very difficult in reality, since the timing of urgent data generation is not generally unknown.

As mentioned in Section 1, the hybrid approach exploits the benefits of both the parametric and channelization-based techniques. Yoon *et al.* [20] suggested the PNP-MAC as a hybrid approach for providing QoS in WBASNs. The PNP-MAC is a TDMA-based MAC that provides QoS in accordance with the priority of traffic. In the PNP-MAC, the data transmit slots (DTSs) are reserved for non-urgent data, while the emergency data transmit slots (ETSs) are used for the transmission of urgent data. The CAP is used for the reservation of the DTSs for non-urgent data and used for the transmission of urgent data. Non-urgent data with higher priority can preempt the DTS reserved for non-urgent data with lower priority. If there is no room for the DTSs, non-urgent data can reserve an ETS *opportunistically*. In other words, non-urgent data perform clear channel assessment (CCA) in the ETS and check for any urgent data transmission with shorter inter-frame space (IFS) and, thus, higher priority than the non-urgent data. Although an emergency alarm can occur during the DTSs, priority is given to non-urgent data in the PNP-MAC. Zhou *et al.* [21] proposed the virtual MAC (V-MAC) for adapting various QoS requirements. The V-MAC is positioned between the network layer and MAC layer and adjusts the data communication between them to satisfy QoS. However, if emergency data are placed into the V-MAC, the data preempt non-time-critical data in the queue. More recently, another hybrid approach has been standardized in IEEE 802.15.6 [23]. Since IEEE 802.15.6 is a merged technology [14,19,20,22,24], it has much in common with the above research. Especially, the methodology of urgent data transmission is mostly adapted from the PNP-MAC [20] and NICT’s MAC proposal [19]. IEEE 802.15.6 uses three different phases of exclusive access phase (EAP), random access phase (RAP) and CAP. The channel access mechanism of high-priority data is fundamentally the same as in the PAP of the NICT’s MAC proposal. Polling access in IEEE 802.15.6 is similar to the ETS reservation in the PNP-MAC in the sense that the polling frame is transmitted.

As we can conclude from the above data, research for WBASNs concentrates on urgent data transmission with a guaranteed time slot for urgent data. However, these research works are not considered to be wasting network utilization caused by the non-transmission of urgent data on the slot. The CoR-MAC, as we mentioned, can resolve this problem with the dual reservation scheme that allows transmitting within unused slots for urgent data.

## 3. Contention over Reservation MAC

In our CoR-MAC, we assume that the WBASN forms a star topology (this is the basic formation of the network; however, this network can be further extended into multihop networks as in IEEE 802.15.6) consisting of one coordinator ($n_{0}$) and *N* sensor nodes ($n_{i}$, *i* ∈ {1, 2, …,N}) that monitor the physiological status of a human body. These nodes can be implantable or wearable devices. Although there are N+1 nodes in the network, we assume there are *m* (m<N+1) nodes that can generate urgent data depending on their detection task. Let U denote the set of such nodes $u_{i}$ where (i∈ {1, 2, …,m}). Further, we assume that the sensor nodes are fixed; hence, there is no mobility in the network. In the network, times are slotted by the coordinator, so that the sensor nodes can be synchronized. Moreover, we assume there is no direct link between sensor nodes, and thus, transmission from one to another has to be relayed by the coordinator. This assumption is because two sensor nodes can be located very far apart, so as not to be in the communication range.

### 3.1. System Model and Basic Assumptions

In the CoR scheme, traffic is divided into three priority levels in descending order of urgency: urgent, time-critical and non-time-critical data. The urgent data include life-threatening conditions of the patient and need to be relayed immediately. The time-critical data include real-time data, such as electrocardiogram (ECG), electroencephalogram (EEG), electromyographic (EMG) and medical video data. The non-time-critical data include messaging and traffic service, as in [12]. Similar to $u_{i}$, we denote $c_{i}$ the node that generates time-critical data. It is possible that a node generates both urgent and time-critical data.

### 3.2. Superframe Structure

**Figure 1:**
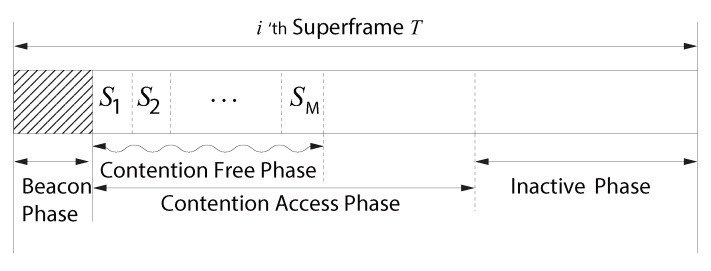
Contention over reservation (CoR)-MAC superframe structure.

To accommodate all three traffic types in Section 3.1, the CoR-MAC employs a superframe structure consisting of the beacon, CFP and CAP, as shown in Figure 1. The three traffic types are transmitted as follows:

1. No non-emergency traffic can be transmitted during an inactive phase that is designed for energy reduction. Although transmission is not allowed, this phase can be utilized for emergency data transmission, as in [4]. Transmission of emergency data during an inactive phase is beyond the scope of this paper.
2. The beacon phase is the time in which only the coordinator is allowed to transmit a beacon frame, and it is used for network synchronization and advertising information, such as time slot allocation. The beacon frame includes superframe length, subsequent superframe start time, beacon length, CFP length, CAP length, length of the CFP time slot, network ID, the number of time slots in the CFP and the CFP allocation map, including slot number and node ID. The size of the beacon is varied according to the size of the CFP allocation map. If the number of nodes is 10∼15, the size of the beacon will be around 228∼278 bits when the size of the CFP allocation information per node is 10 bits (five bits for the number of slots and five bits for the node’s ID).
3. Urgent data can be transmitted during either the CFP or CAP. A detailed description of urgent data transmission is described below.
4. The time-critical data can be transmitted during CAP or the reserved time slot in CFP, as long as urgent data are not being transmitted.
5. Non-time-critical data can be transmitted during either CFP or CAP, as long as urgent, time-critical or other non-time-critical data are not being transmitted.

Generally, transmitting urgent data over a CAP is not desirable, since timely transmission is not guaranteed due to contentions. Therefore, it would be better to transmit urgent data over a CFP by reserving a dedicated slot. However, the dedicated time slot might not be frequently utilized since urgent traffic is generated sporadically in general, resulting in degraded throughput. Therefore, in the CoR-MAC, the CFP is divided into time slots $S_{1}$, $S_{2}$, *…*, $S_{M}$, and $u_{i}$ is allocated to $S_{i}$ (i∈ {1, 2, …,M}). Initially (M=0), there is no CFP, and the CAP spans from the end of the beacon phase to the beginning of the inactivity phase. When $u_{i}$ joins the network, the CFP expands, while the CAP shrinks. This join request for $u_{i}$ is transmitted over the CAP. *M* is the maximum allowable number of time slots in the CFP and is selected based on the number of nodes that generate urgent data. We call this phase the *self* CFP (SCFP). Further, to increase the transmission opportunities, urgent data of $u_{i}$ can be transmitted over $S_{j}$ (j≠i) if $S_{j}$ is not used for $u_{j}$. Unlike the SCFP, we call this phase the *opportunistic* CFP (OCFP). To prioritize $u_{j}$ against $u_{i}$ over $S_{j}$, $u_{j}$ transmits its urgent data at the beginning of the slot boundary, while $u_{i}$ waits for short inter-frame space (SIFS) before transmitting a frame.

Moreover, in the CoR-MAC, $S_{i}$ can be used for the transmission of other traffic types as long as urgent data from $u_{i}$ or $u_{j}$ (j≠i) is not being transmitted over $S_{i}$. This is because urgent traffic is sporadically generated, and $S_{i}$ can be wasted as described in the above. Therefore, $S_{i}$’s can be reserved (this is why our scheme is referred to as “dual reservation,” *i.e.*, $S_{i}$ can be reserved by both urgent and time-critical data) for time-critical data or used for transmission of non-time-critical data. Of course, the reservation of $S_{i}$’s for time-critical data is performed at the CAP, *i.e*., a reservation request command for time-critical data is transmitted over the CAP. To prioritize urgent data against time-critical data, time-critical data wait for the medium inter-frame space (MIFS) before transmitting a frame, where MIFS > SIFS. In other words, even if time-critical data are reserved at $S_{i}$, urgent data from $u_{j}$ (∀j) can take $S_{i}$. In order to reduce collisions among $u_{j}$ (j≠i), a contention window is used in the range of (0, $U_{\max}$), where $U_{\max}<$ MIFS-SIFS. Likewise, to prioritize time-critical data against non-time-critical data, non-time-critical data wait for a long inter-frame space (LIFS) before transmitting a frame, where LIFS > SIFS.

In addition to the CFP, the CAP is used for the transmission of urgent data and non-time-critical data. In this way, urgent data have ample opportunity to access a channel. Using this mechanism, our scheme can transmit the urgent data significantly faster than the method in [20].

### 3.3. Protocol Description

**Figure 2:**
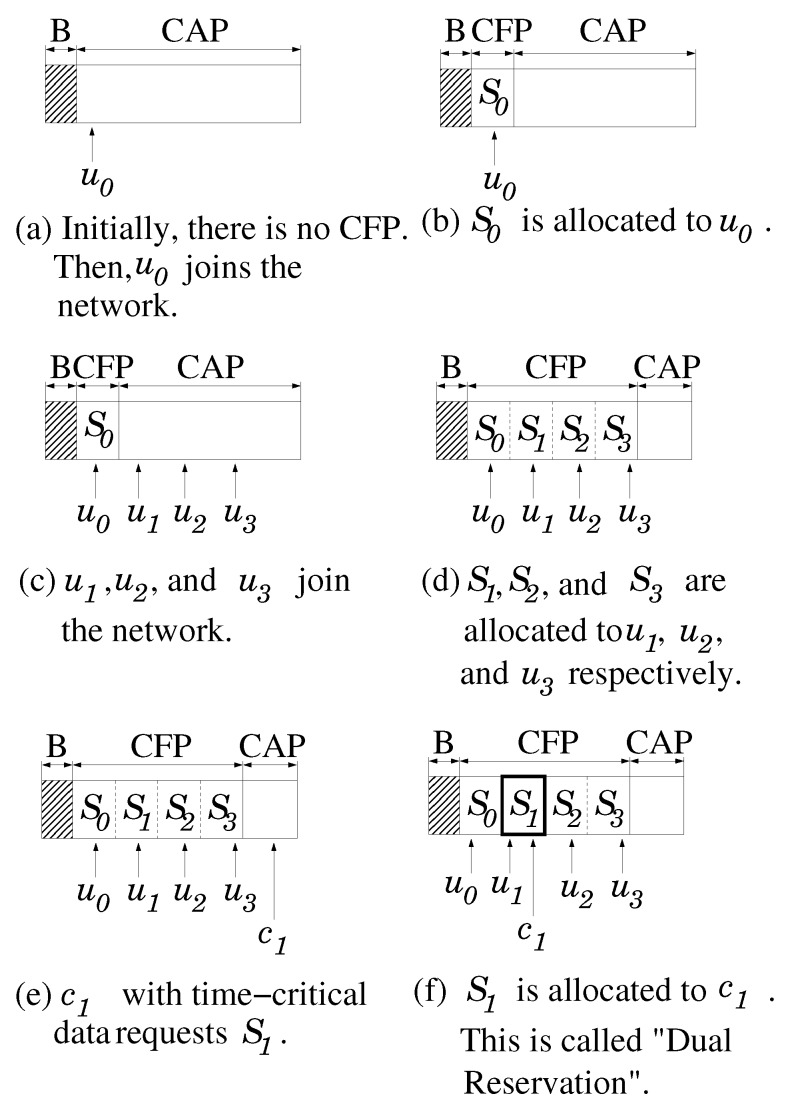
Dual reservation process of the CoR-MAC (B denotes beacon). CAP, contention access phase; CFP, contention-free phase.

Initially, there is no CFP, and the CAP is extended to the end of the beacon phase, as shown in Figure 2a. Suppose that $u_{0}$ joins the network. Then, $S_{1}$ is allocated to $u_{1}$, as shown in Figure 2b. Now, suppose $u_{2}$, $u_{3}$ and $u_{4}$ join the network (Figure 2c). $S_{2}$, $S_{3}$ and $S_{4}$ are allocated to $u_{2}$, $u_{3}$ and $u_{4}$, respectively, as shown in Figure 2d. Moreover, we assume $c_{1}$ has time-critical data to transmit and, so, requests a time slot as, shown in Figure 2e. After receiving this request, the coordinator allocates $S_{1}$ for $c_{1}$, as shown in Figure 2f. Note that $S_{1}$ is reserved for both $u_{1}$ and $c_{1}$. This is the reason why we call the scheme dual reservation.

The CoR-MAC algorithm for the coordinator is shown in Algorithm 1. When $u_{i}$ or $c_{i}$ joins the network, the coordinator determines the number of time slots in the CFP. If the number is less than *M*, the coordinator allocates $S_{i}$ to the $u_{i}$ or $c_{i}$. Otherwise, the coordinator rejects the join request (Lines 5–12). Moreover, if the current time is within the beacon phase, the coordinator sends a beacon frame along with the superframe length, the subsequent superframe start time, the beacon length, the CFP length, the CAP length, the length of the CFP time slot, the network ID, the number of time slots in the CFP and the CFP allocation map, including the slot number and node ID (Lines 2–4). If data and the RTSare received from $n_{i}$, the coordinator sends ACK and CTS, respectively (Lines 13–17).

**Algorithm 1** The CoR-MAC algorithm for the coordinator.
 1:$u_{i}$← The node that generates urgent data; 2:$c_{i}$← The node that generates time-critical data; 3:*M*← The maximum allowable number of time slots in CFP; 4:$S_{i}$← The slot that is located at the *i*’-th position; 5:$n_{i}$← The node whose ID is *i*; 6:**loop** 7: **if** Current time = start of beacon phase **then** 8:  Send beacon with CFP allocation map 9: **end if**10: **if** $u_{i}$ or $c_{i}$ joins the network **then**11:  **if** # of time slots in the CFP < *M* **then**12:   Allocate $S_{i}$ to the $u_{i}$ or $c_{i}$13:   Update CFP allocation map14:  **else**15:   Reject the join request16:  **end if**17: **end if**18: **if** DATA is received from $n_{i}$ **then**19:  Send ACK to $n_{i}$ after SIFS20: **else** **if** RTS is received from $n_{i}$ **then**21:  Send CTS to $n_{i}$ after SIFS22: **end if**23:**end loop**

**Algorithm 2** The CoR-MAC algorithm for the node.
 1:$S_{i}$← The slot which is located in the *i*’-th position; 2:Φ ← The current time; 3:$\Phi_{scfp}$← The phase of SCFP; 4:$\Phi_{ocfp}$← The phase of OCFP; 5:$\Phi_{cap}$← The phase of CAP; 6:**loop** 7: **if** BEACON is received from the coordinator **then** 8:  Synchronize itself to the coordinator; 9:  Record this $S_{i}$ based on CFP allocation map;10: **else** **if** The node has urgent data to transmit **then**11:  **if** Φ ⊂ $\Phi_{scfp}$ **then**12:   Send urgent DATA immediately;13:  **else** **if** Φ ⊂ $\Phi_{ocfp}$ **then**14:   Send DATA with CSMA/CA after SIFS;15:  **else** **if** Φ ⊂ $\Phi_{cap}$ **then**16:   Send DATA with CSMA/CA;17:  **end if**18: **else** **if** The node has time-critical data to transmit **then**19:  **if** Φ ⊂ $(\Phi_{scfp}$ ∪ $\Phi_{ocfp})$ **then**20:   Send DATA after MIFS;21:  **else** **if** Φ ⊂ $\Phi_{cap}$ **then**22:   Send DATA with CSMA/CA;23:  **end if**24: **else** **if** The node has non-time-critical data to transmit **then**25:  **if** Φ ⊂ $(\Phi_{scfp}$ ∪ $\Phi_{ocfp})$ **then**26:   Send DATA with CSMA/CA after LIFS;27:  **else** **if** Φ ⊂ $\Phi_{cap}$ **then**28:   Send DATA with CSMA/CA;29:  **end if**30: **end if**31:**end loop**

The CoR-MAC algorithm for the sensor node is shown in Algorithm 2. When a beacon is received from the coordinator, nodes synchronize themselves to the coordinator and record this $S_{i}$ based on the CFP allocation map (Lines 2–4). If the node has urgent data and the current time is within the SCFP, the data are transmitted immediately (Lines 6–7). If the current time is within the OCFP, the node sends the urgent data with CSMA/CA after SIFS (Lines 8–9). However, the node sends the urgent data with CSMA/CA if the current time is within CAP (Lines 10–11). On the other hand, if the node has time-critical data to transmit and the current time is within the CFP, the node sends its data after MIFS (Lines 14–15). If the current time is within the CAP, the node sends its data with CSMA/CA (Lines 16–17). Lastly, if the data are non-time-critical and the current time is within CFP, the node sends its data after LIFS (Lines 20–21). If the current time is within CAP, the node sends its data with CSMA/CA (Lines 22–23). During CSMA/CA, the backoff counter is decreased as long as the channel is idle, frozen when the channel is busy and reactivated when the channel is idle in the CAP or OCFP. If there is no other transmission when the backoff counter reaches zero, the node will transmit its data. To achieve energy efficiency, a node that could not transmit data in a timeslot should go to inactive mode until the next beacon frame in CoR-MAC.

## 4. Theoretical Approach

In this section, we analyze an urgent data delay of the proposed scheme with a theoretical approach. We show that our scheme guarantees the delay requirement of the urgent data. In the following, we describe our assumption and system model to obtain the expected delay in Section 4.1. Based on the assumption, we derive the expected delay of the proposed scheme in Section 4.2. The meanings of the variables in theoretical approach are summarized in Table 1.

### 4.1. System Model

We assume that each node generates both urgent data and non-urgent data. Note that the arrival rates of urgent data follow exponential distributions with $\lambda_{u}$ and the arrival rates of non-urgent data with $\lambda_{n}$. We assume that the allowable number of time slots in the CFP (denoted as *M*) is less than or equal to the number of nodes *N*. In the CoR-MAC protocol, non-urgent data can be transmitted when no node has urgent data in CFP. Therefore, we do not consider non-urgent data in CFP. Denoting $\zeta_{\tau_{cfp}}$ as the probability that a node does not generate urgent data in the CFP slot, $\zeta_{\tau_{cfp}}$ can be described as:

(1) $$\begin{array}{c} \zeta_{\tau_{cfp}}=e^{-\lambda_{u}\cdot \tau_{cfp}} \end{array}$$

where $\tau_{cfp}$ is the length of a CFP slot.

We assume that CAP consists of a number of system time slots ($\tau_{cap}$). A system time slot is a very short amount of time, such as a system tick. We denote *L* as the number of $\tau_{cap}$ in a $T_{CAP}$. In CAP, however, urgent data exist among other urgent data and other non-urgent data, although urgent data have higher priority than non-urgent data. Moreover, a transmission can be either a new transmission or a retransmission. We denote *G* as the rate of attempted transmissions, including retransmission, new arrivals, urgent data and non-urgent data. Although *G* depends on time in reality, we assume that *G* is a constant value in our analysis.

Denoting $\zeta_{\tau_{cap}}$ as the probability that a node can transmit by CSMA/CA at a time unit ($\tau_{cap}$= one system time slot) in the CAP, $\zeta_{\tau_{cap}}$ can be described as:

(2) $$\begin{array}{c} \zeta_{\tau_{cap}}=e^{-\left(N-1\right)G\cdot \tau_{cap}} \end{array}$$

We denote the probability of transmission failure due to errors or collision and that of transmission success as $P_{f}$ and $P_{s}$, respectively. $P_{s}$ is described as $P_{s}=1-P_{f}$.

Let $P_{w}$ be the probability that a node can occupy the channel to contendamong other nodes, then $P_{w}$ can be obtained by:

(3) Pw=∑k=0N-1(1-ζτcfp)k·ζτcfp(N-k-1)·1k+1·N-1k

where *N* is the number of nodes and *k* is the number of nodes with urgent data. In the CoR protocol, each node has the opportunity to transmit data in CAP and CFP. CFP consists of dedicated slots for each user, and we describe a user who allocates the slot as an owner.

### 4.2. Expected Delay of Urgent Data

We denote the expected delay from running time *t* when *t* is located at Phase(∈{OCFP,SCFP,CAP}) in the *i*-th superframe as $D\;(Phase_{i}^{t})$.

**Figure 3:**
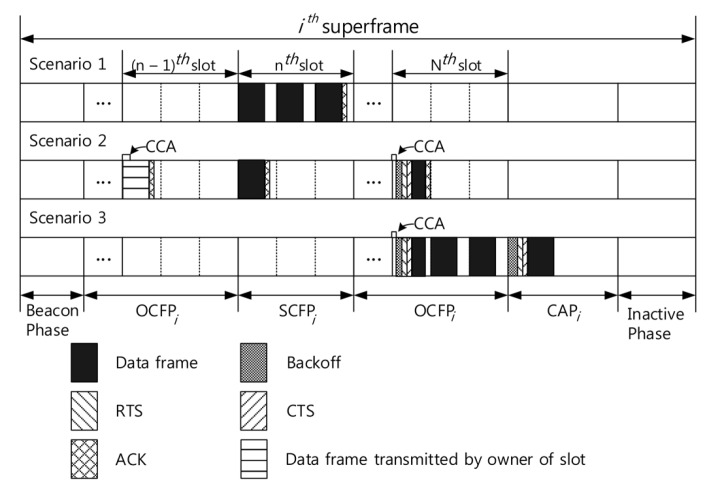
Channel occupancy scenarios of the urgent data. CCA, clear channel assessment; OCFP, opportunistic CFP; SCFP, self CFP.

In the CoR-MAC protocol, there are three channel occupancy scenarios for urgent data, as shown in Figure 3. In SCFP, a node can occupy the CFP slot without contention if the node has urgent data. This is because SCFP is allocated to the node. As shown in the figure, the node has a dedicated slot with three transmit chances in SCFP. If the transmissions are successful in the slot, then the delay is $\tau_{cfp}$. If transmissions fail in the slot, however, there are two scenarios of going to CAP or to OCFP after time $\tau_{cfp}$. Since the number of nodes is *N*, the probability of transmission to OCFP after SCFP is (N-1)/N, and the probability of transmission to CAP is 1/N. Therefore, the expected delay $D(SCFP_{i}^{t})$ can be obtained by:

(4) $$\begin{array}{cc} D(SCFP_{i}^{t})≃ & \left(1-P_{f}^{3}\right)\cdot \tau_{cfp}+\\ & P_{f}^{3}\left(\tau_{cfp}+\frac{D(CAP_{i}^{t})}{N}+\frac{N-1}{N}D\left(OCFP_{i}^{t}\right)\right) \end{array}$$

where $D(CAP_{i}^{t})$ is the expected delay in CAP and $D(OCFP_{i}^{t})$ is the expected delay in OCFP.

The OCFP slot is dedicated to another node (called the slot owner). Therefore, each node has an opportunity to transmit if the slot owner does not have urgent data. If the slot owner has urgent data, the other nodes wait $\tau_{cfp}$ and choose one of the three scenarios of transmission to CAP, OCFP or SCFP. This scenario is described at the n-1’-th slot in Figure 3, Scenario 2. The probabilities of transmission to CAP, OCFP, SCFP are 1/N, (N-2)/N, 1/N, respectively.

If the slot owner of the slot does not have urgent data, each node with urgent data vies with other nodes. If a node wins contention and transmissions are successful in the slot, then the delay is $\tau_{cfp}$. This scenario is described at the *N*’-th slot in Figure 3, Scenario 2. If a node loses contention or transmissions fail in the slot, however, transmission occurs in CAP, OCFP or SCFP after time $\tau_{cfp}$. Therefore, the expected delay $D(OCFP_{i}^{t})$ can be obtained by Equation (5), where $P_{s}$, $P_{f}$ and $P_{w}$ are probabilities described in Section 4.1.

(5) $$\begin{array}{cc} D(OCFP_{i}^{t})≃ & \left(1-\zeta_{\tau_{cfp}}\right)(\tau_{cfp}+\frac{D(SCFP_{i}^{t})}{N}+\frac{D(CAP_{i}^{t})}{N}+\frac{N-2}{N}D\left(OCFP_{i}^{t}\right))+\\ & \zeta_{\tau_{cfp}}(P_{w}(P_{s}\cdot \tau_{cfp}+P_{f}(\tau_{cfp}+\frac{D(SCFP_{i}^{t})}{N}+\frac{D(CAP_{i}^{t})}{N}+\frac{N-2}{N}D\left(OCFP_{i}^{t}\right))+\\ & \left(1-P_{w}\right)\left(\tau_{cfp}+\frac{D(SCFP_{i}^{t})}{N}+\frac{D(CAP_{i}^{t})}{N}+\frac{N-2}{N}D\left(OCFP_{i}^{t}\right)\right)) \end{array}$$

CAP consists of a number of $\tau_{cap}$. We described $\tau_{cap}$ as the duration of one system time slot in Section 4.1, and *L* is the number of $\tau_{cap}$ in CAP (e.g., the length of CAP is $\tau_{cap}\times L$). If a node cannot transmit with probability $1-\zeta_{\tau_{cap}}$, then the node waits $\tau_{cap}$ and transmits during CAP or the next superframe. The probabilities of transmission during CAP or the next superframe are $(L-T_{send}/\tau_{cap})/L$ and $\frac{T_{send}}{\tau_{cap}L}$, respectively. If a node transmits in the CAP and the transmissions are successful, then the delay is $T_{send}=(RTStime$+CTStime+Datatime+ACKtime). This scenario is described in Figure 3, Scenario 3. Therefore, the expected delay $D(CAP_{i}^{t})$ can be obtained by Equation (6).

(6) $$\begin{array}{cc} D(CAP_{i}^{t})≃ & \zeta_{\tau_{cap}}(P_{s}\cdot T_{send}+P_{f}(\tau_{cap}+\;\frac{L-T_{send}/\tau_{cap}}{L}D\left(CAP_{i}^{t}\right)+\\ & \frac{T_{send}}{\tau_{cap}L}(T_{i}+T_{b}\;+\frac{D(SCFP_{i}^{t})}{N}+\frac{N-1}{N}D\left(OCFP_{i}^{t}\right)))+\\ & \left(1-\zeta_{\tau_{cap}}\right)\left(\tau_{cap}+\frac{T_{send}/\tau_{cap}}{L}D\left(CAP_{i}^{t}\right)\right)+\frac{T_{send}}{\tau_{cap}L}\left(T_{i}+T_{b}+\frac{D(SCFP_{i}^{t})}{N}+\frac{N-1}{N}D\left(OCFP_{i}^{t}\right)\right)) \end{array}$$

We use Gaussian elimination to solve these trinomial expressions. To use Gaussian elimination, each equation is arranged with unknown quantities ($D(SCFP_{i}^{t})$, $D(OCFP_{i}^{t})$, $D(CAP_{i}^{t})$), as shown in Equations (7)–(9), respectively.

(7) $$\begin{array}{c} D\left(SCFP_{i}^{t}\right)≃\tau_{cap}+P_{f}^{3}\left(\frac{D(CAP_{i}^{t})}{N}+\frac{\left(N-1\right)D\left(OCFP_{i}^{t}\right)}{N}\right) \end{array}$$

(8) $$\begin{array}{c} D\left(OCFP_{i}^{t}\right)≃\left(\tau_{cfp}+\left(1-P\left(t\right)P_{w}P_{s}\right)\left(\frac{D(SCFP_{i}^{t})}{N}+\frac{D(CAP_{i}^{t})}{N}\right)\right)\cdot \frac{1}{1-\left(1-P\left(t\right)P_{w}P_{s}\right)\frac{N-2}{N}} \end{array}$$

(9) $$\begin{array}{cc} D(CAP_{i}^{t})≃ & (-T_{send}\left(\zeta_{\tau_{cap}}\cdot P_{f}-\zeta_{\tau_{cap}}+1\right)\cdot \left(D\left(SCFP_{i}^{t}\right)+\left(N-1\right)D\left(OCFP_{i}^{t}\right)\right)+\\ & \zeta_{\tau_{cap}}(\tau_{cap}\left(P_{f}-1\right)+P_{s}\cdot T_{send}-T_{send}\left(T_{i}+T_{b}\right)/\left(\tau_{cap}\cdot L\right)+\tau_{cap})/\\ & \left(N\cdot \tau_{cap}\cdot \left(\zeta_{\tau_{cap}}\cdot P_{f}\left(L-T_{send}/\tau_{cap}\right)+T_{send}/\tau_{cap}-\zeta_{\tau_{cap}}\cdot T_{send}/\tau_{cap}-L\right)\right) \end{array}$$

To simplify the equation, we use auxiliary variables *α*, *β*, *γ*, *δ* and *ϵ* to construct the augmented matrix for Gaussian elimination.

(10) $$\begin{array}{c} \alpha =-\frac{P_{f}^{3}}{N} \end{array}$$

(11) $$\begin{array}{c} \beta =-\frac{1-P\left(t\right)P_{w}P_{s}}{(1-\left(1-P\left(t\right)P_{w}P_{s}\right)\frac{N-2}{N})N} \end{array}$$

(12) $$\begin{array}{c} \gamma =\frac{\tau_{cfp}}{1-\left(1-P\left(t\right)P_{w}P_{s}\right)\frac{N-2}{N}} \end{array}$$

(13) $$\begin{array}{c} \delta =\frac{T_{send}\left(\zeta_{\tau_{cap}}\cdot P_{f}-\zeta_{\tau_{cap}}+1\right)}{\left(N\cdot \tau_{cap}\cdot (\zeta_{\tau_{cap}}\cdot P_{f}\left(L-\frac{T_{send}}{\tau_{cap}}\right)+\frac{T_{send}\left(1-\zeta_{\tau_{cap}}\right)}{\tau_{cap}}-L)\right)} \end{array}$$

(14) $$\begin{array}{c} \epsilon =\frac{\left(\zeta_{\tau_{cap}}(\tau_{cap}P_{f}+P_{s}T_{send}-\frac{T_{send}\left(T_{i}+T_{b}\right)}{\tau_{cap}L}\right)}{\left(N\tau_{cap}(\zeta_{\tau_{cap}}P_{f}\left(L-\frac{T_{send}}{\tau_{cap}}\right)+\frac{T_{send}\left(1-\zeta_{\tau_{cap}}\right)}{\tau_{cap}}-L)\right)} \end{array}$$

Using these auxiliary variables, the augmented matrix can be obtained as:

(15) $$\left.\begin{array}{cc} \left[\begin{array}{ccc} 1 & (N-1)\alpha & \alpha \\ \beta & 1 & \beta \\ \delta & (N-1)\delta & 1 \end{array}\right| & \begin{array}{c} \tau_{cfp}\\ \gamma \\ \epsilon \end{array} \end{array}\right].$$

Utilizing the augmented Matrix (15) for Gaussian elimination, we get Equations (16)–(18).

(16) $$\begin{array}{cc} D(CAP_{i}^{t})≃ & \frac{\left(\left(\epsilon -\delta \tau_{cfp}\right)-\left(\frac{\gamma -\beta \tau_{cfp}}{1-(N-1)\alpha \beta }\right)\cdot \left(\left(N-1\right)\left(1-\alpha \right)\delta \right)\right)}{\left(\left(1-\alpha \delta \right)-\left(\frac{\beta -\alpha \beta }{1-(N-1)\alpha \beta )}\right)\left(\left(N-1\right)\left(1-\alpha \right)\delta \right)\right)} \end{array}$$

(17) $$\begin{array}{c} D\left(OCFP_{i}^{t}\right)≃\frac{\gamma -\beta \tau_{cfp}}{1-(N-1)\alpha \beta }-\frac{\beta -\alpha \beta }{1-(N-1)\alpha \beta }D\left(CAP_{i}^{t}\right) \end{array}$$

(18) $$\begin{array}{c} D\left(SCFP_{i}^{t}\right)≃\tau_{cfp}-\left(N-1\right)\alpha D\left(OCFP_{i}^{t}\right)-\alpha D\left(CAP_{i}^{t}\right) \end{array}$$

Substituting each equation, we obtain the values of $D(CAP_{n}^{t})$, $D(OCFP_{n}^{t})$ and $D(SCFP_{n}^{t})$.

Data generation follows an exponential distribution; this is contrary to our expectation. Therefore, the delay associated with each type of data is obtained using Equation (19), where $T_{sf}$ is the length of a superframe.

(19) $$\begin{array}{cc} E[Delay]≃ & \frac{\tau_{cfp}}{T_{sf}}\cdot D\left(SCFP_{i}^{t}\right)+\frac{\left(N-1\right)\tau_{cfp}}{T_{sf}}\cdot D\left(OCFP_{i}^{t}\right)+\frac{L\cdot \tau_{cap}}{T_{sf}}\cdot D\left(CAP_{i}^{t}\right)+\\ & \frac{T_{i}}{T_{sf}}\left(T_{i}+T_{b}+\frac{D(SCFP_{i}^{t})}{N}+\frac{\left(N-1\right)D\left(OCFP_{i}^{t}\right)}{N}\right)+\\ & \frac{T_{b}}{T_{sf}}\left(T_{b}+\frac{D(SCFP_{i}^{t})}{N}+\frac{\left(N-1\right)D\left(OCFP_{i}^{t}\right)}{N}\right) \end{array}$$

**Figure 4:**
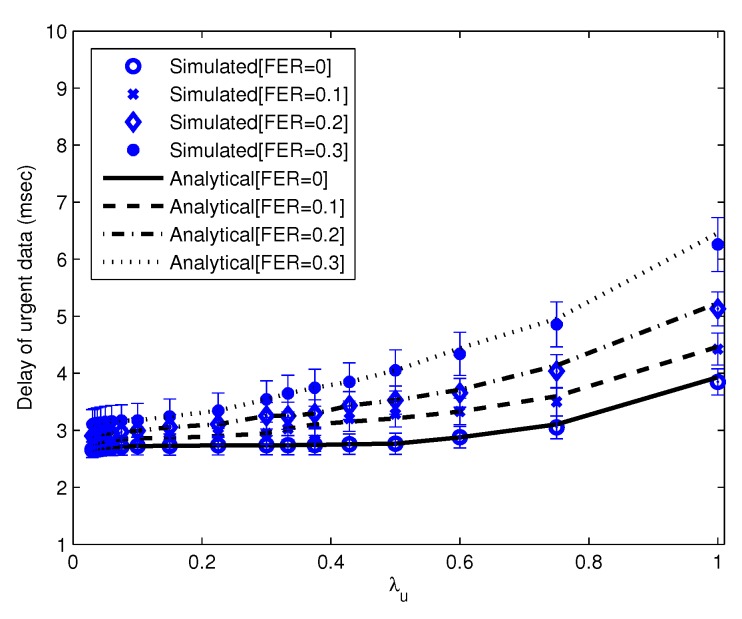
The average delay of urgent data *vs*. $\lambda_{u}$. ($\lambda_{n}$ = 1 frames/s per node).

**Figure 5:**
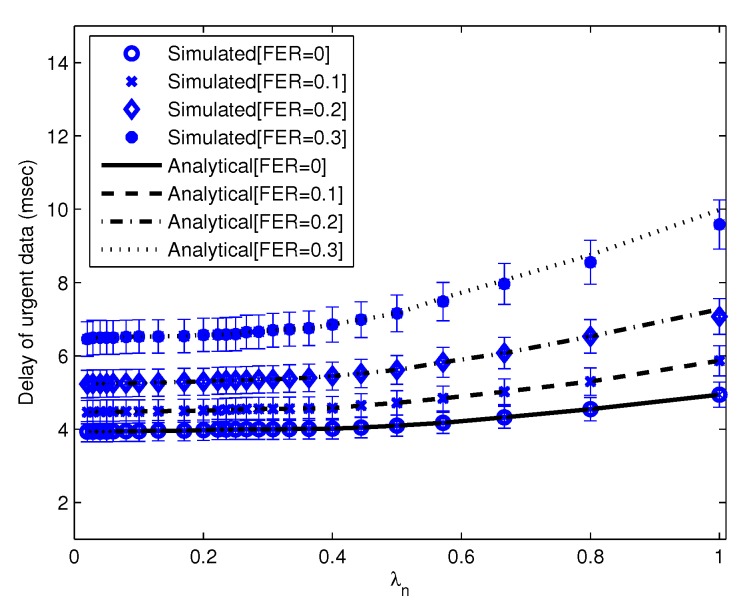
The average delay of urgent data *vs*. $\lambda_{n}$. ($\lambda_{u}$ = 66.6 frames/s per node). SIFS, short inter-frame space; MIFS, medium IFS; LIFS, long IFS.

Using parameters listed in Table 2 with 20 nodes, we compare the results of our theoretical model to the simulation. We show that the average delay of urgent data *vs*. $\lambda_{u}$($\lambda_{n}$ = 1) is almost similar between the theoretical model and simulation, as shown in Figure 4. We also show in Figure 5 that the average delay of urgent data *vs*. $\lambda_{n}$($\lambda_{u}$ = 66.6) using a theoretical model is similar to the simulated delay.

## 5. Performance Evaluation

In this section, we evaluate the performance of the proposed protocol compared to those of IEEE 802.15.4 and IEEE 802.15.6. Much research focused on WBASNs is based on IEEE 802.15.4 technology [7,8,9,10,11]. Furthermore, IEEE 802.15.6 uses a hybrid approach that exploits the benefits of both parametric- and channelization-based techniques.

For performance evaluation, we use the OPNET modeler [25] and record data transmission information during a one-hour simulation time. In the simulation, we form a star topology where the coordinator and sensor nodes are located within a 1-m range. In the given topology, the positions of sensor nodes are chosen randomly. The simulation parameters are listed in Table 2.

In this simulation, we measure the average delay of the urgent, time-critical and non-time-critical data; the utilization in CAP and CFP; the average aggregate throughput of three traffic types (urgent, time-critical, non-time-critical); and the energy consumption.

### 5.1. Delay

**Figure 6:**
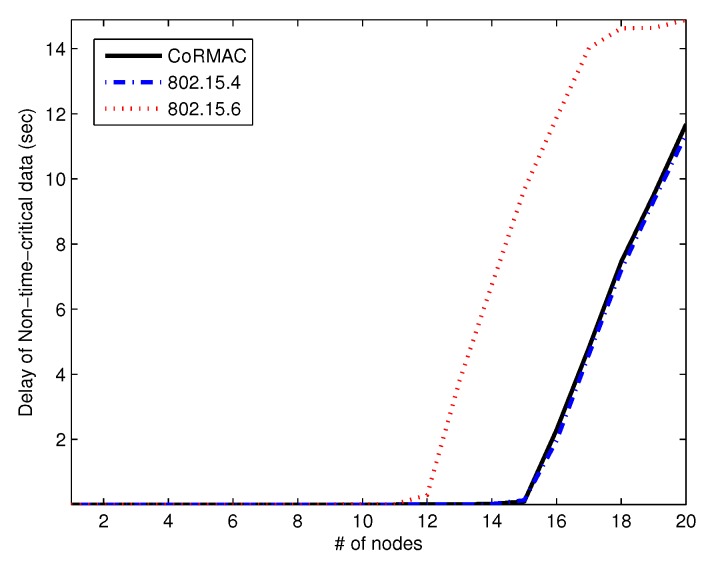
The average delay of non-time-critical data *vs.* the number of nodes.

Figure 6 shows the average non-time-critical data delay *versus* the arrival rate of the number of nodes. The delay of IEEE 802.15.6 increases from 11–12 nodes since IEEE 802.15.6 has an exclusive access phase (EAP) for the processing of urgent data and time-critical data. The EAP is efficient for processing urgent data and time-critical data, but cannot process non-time-critical data. In addition, the CoR-MAC and IEEE 802.15.4 can process non-time-critical data during the entire superframe. With a high arrival rate, the non-time-critical data delay of all schemes increases extremely, since those schemes have lower priority of non-time-critical data, and those MACs are unable to process non-time-critical data.

**Figure 7:**
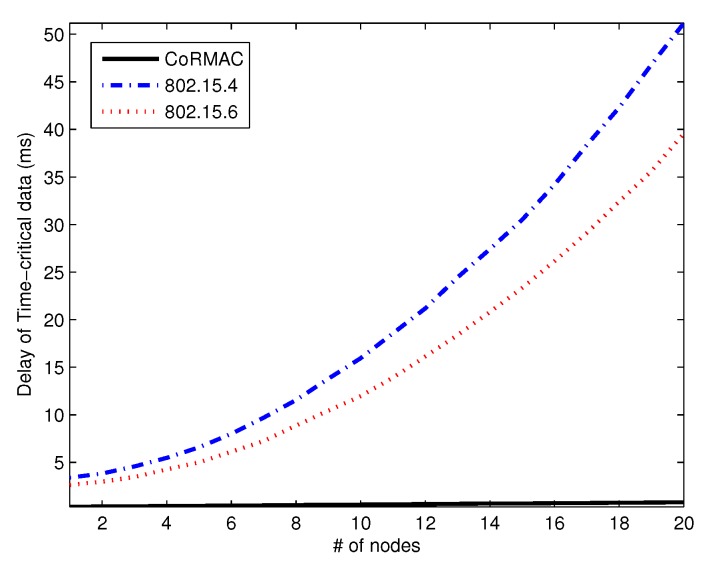
The average delay of time-critical data *vs*. the number of nodes.

**Figure 8:**
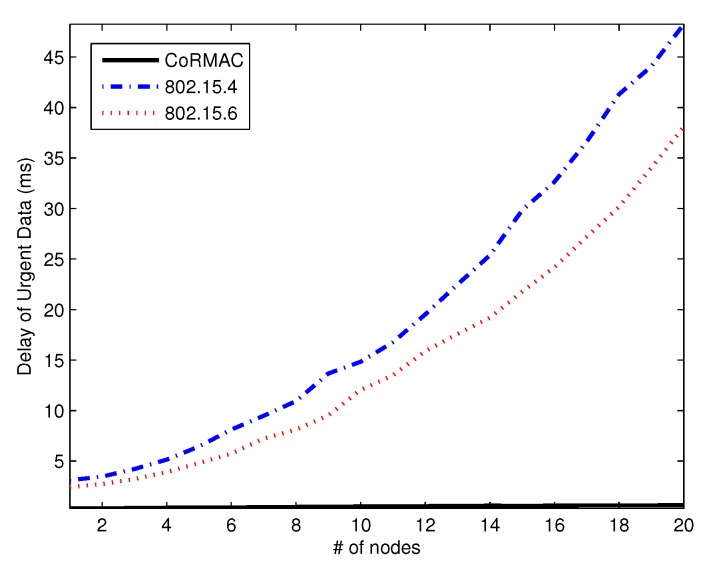
The average delay of urgent data *vs.* the number of nodes.

Figure 7 and Figure 8 show the average time-critical data and urgent data delay *versus* the number of nodes, respectively. The delay of IEEE 802.15.4 is higher than that of the other schemes because IEEE 802.15.6 has EAP. Furthermore, CoR-MAC has a dual reservation scheme for urgent data and time-critical data. In any environment, CoR-MAC has a shorter delay than the other schemes, since the dual reservation scheme allows use of a slot even if the slot is owned by another node. IEEE 802.15.6 and IEEE 802.15.4 also have reserved slots; however, in these schemes, a slot cannot be used if it is owned by another node.

For urgent data, the goal is to guarantee a delay that is below a given deadline (20 ms), not that the delay is the minimum possible value. Therefore, we measure the percentage of packets that exceeds the required deadline. Table 3 shows the delay statistics of urgent data with 15 nodes. As shown in the table, CoR-MAC has the best performance in terms of minimum, maximum and average delays, as well as the percentage over the deadline. Noticeably, 32.17% and 13.17% of urgent data in IEEE 802.15.4 and IEEE 802.15.6 cannot be transmitted by the deadline, respectively. However, in the CoR-MAC, urgent data transmission can meet the deadline with 100%. The CoR-MAC only guarantees the deadline of emergency transmission among the three schemes.

### 5.2. Throughput

**Figure 9:**
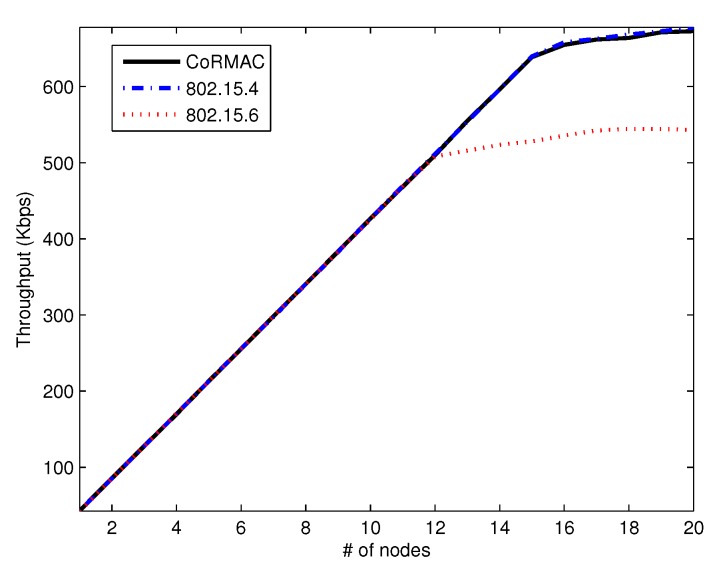
The average throughput of data *vs.* the number of nodes.

The throughput is measured by the sensor nodes. When a node receives an ACK message from a data frame, it records the throughput with the size of the data frame. Figure 9 shows the aggregate throughput of data. The throughput of IEEE 802.15.6 is lower than that of others with a high arrival rate because IEEE 802.15.6 has EAP, which cannot transmit low-priority data. Since IEEE 802.15.4 can transmit non-time-critical data in the whole region, like the CoR-MAC, the throughputs of these schemes are very similar.

### 5.3. Utilization

**Figure 10:**
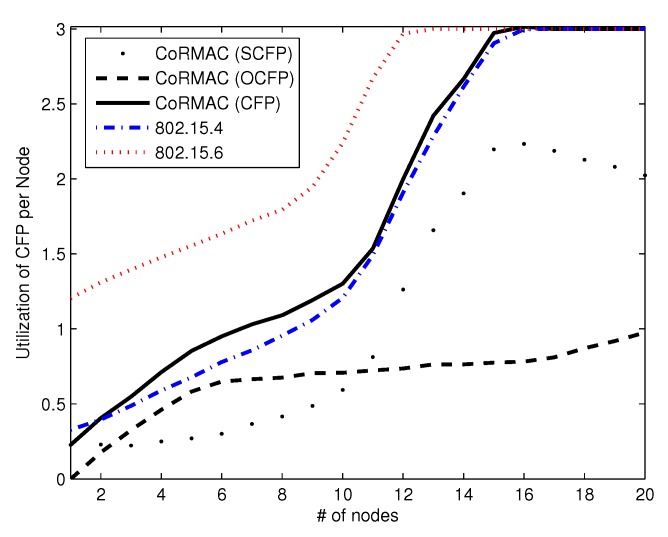
The utilization in CFP *vs.* the number of nodes.

The utilization of a phase is measured by a node. When the node successfully transmits a data frame, the node records a utilization in that phase. Figure 10 shows the average number of transmitted packets with dual reservation in one beacon frame per node. From the figure, we observe that the utilization of OCFP increases with the number of nodes. If the number of nodes decreases, fewer slots are used by other nodes, because data can be transmitted in SCFP or CAP instead of OCFP. Otherwise, as the number of nodes in OCFP increases, the utilization of OCFP also increases. With 6–16 nodes, the increase in utilization is slower; after 16 nodes, the utilization increases more quickly. This phenomenon is related to the number of nodes in OCFP. When the arrival rate and the number of OCFP are balanced, the utilization of OCFP is relatively constant. However, when most of the superframe contains OCFP, a node that has urgent data or time-critical data can use OCFP with high probability. For this reason, the utilization of OCFP increases with more than 16 nodes. The figure shows a special pattern in CoR-MAC in which the utilization in SCFP is lower than those of the other schemes. This phenomenon is caused by the dual reservation scheme. The dual reservation scheme can decrease the utilization of SCFP by utilizing OCFP. This low utilization of SCFP indicates the success of the dual reservation scheme.

**Figure 11:**
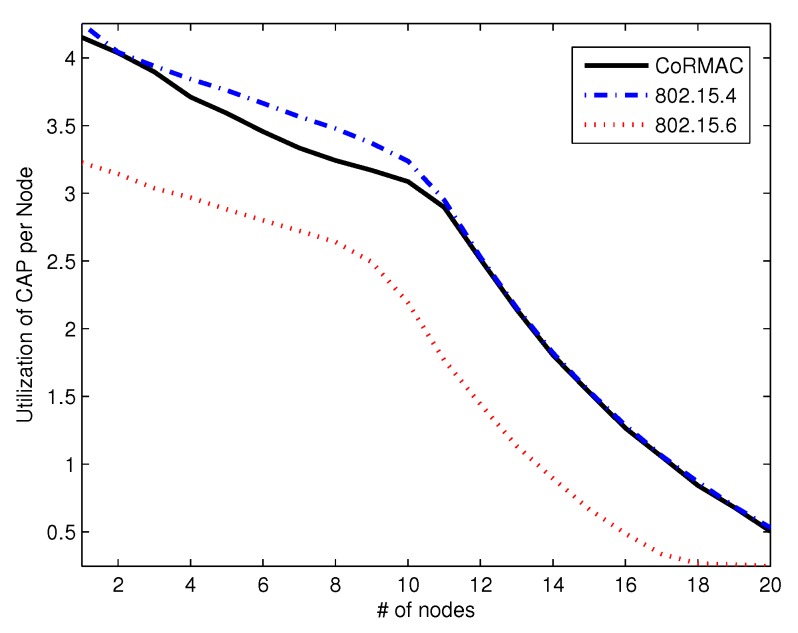
The utilization in CAP *vs.* the number of nodes.

Figure 11 shows the utilization of CAP. The utilization in CAP is like that of the IEEE 802.15.4. This phenomenon shows that the dual reservation scheme does not effect CAP.

### 5.4. Power Consumption

**Figure 12:**
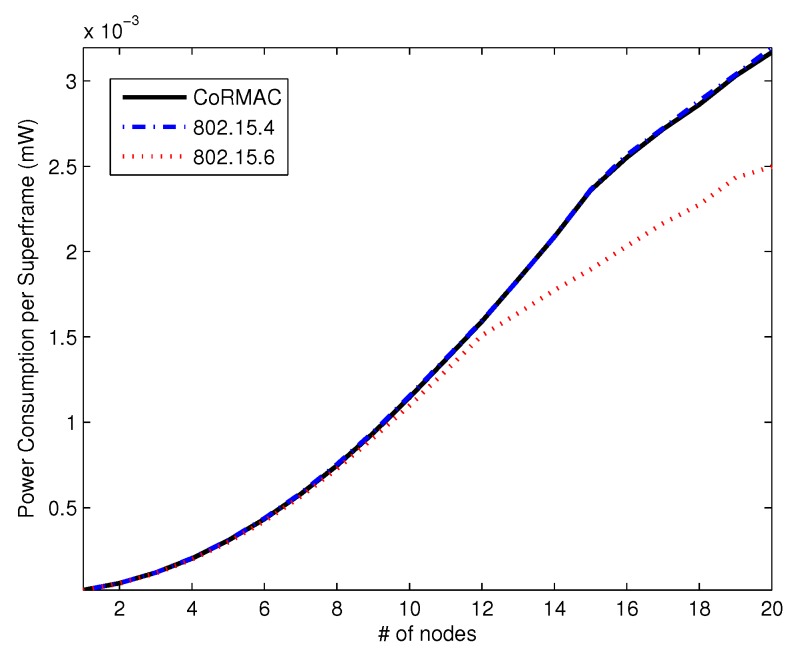
The average power consumption per superframe *vs.* the number of nodes.

Figure 12 shows the average power consumption per superframe. The power consumption of IEEE 802.15.6 is lower than that of the others with a high arrival rate, since IEEE 802.15.6 has EAP, which cannot transmit low-priority data; like throughput.

**Remark 1.**  As previously mentioned, the contention window of CoR-MAC is divided into SIFS, MIFS and LIFS. This will lead to overhead, such as the increasing of each time slot’s size and extra energy consumption. As shown in Figure 12, CoR-MAC consumes a little more power than IEEE 802.15.6. However, the total energy consumption of CoR-MAC is similar to the IEEE 802.15.4. This is because the node enters an inactivity mode when the node cannot transmit the SCFP or OCFP in CoR-MAC.

## 6. Conclusions

In this paper, we addressed the problem of the delay of urgent data and time-critical data. We also addressed the utilization of dedicated slots. Existing WBASN schemes cannot properly resolve the above problems and do not address heavy traffic load situations, since they do not consider urgent data transmission. To reduce the delay of urgent data transmission, we proposed a contention over reservation MAC protocol for WBASNs. Our protocol exploits CFP reservation among nodes. For the reservation, traffic is divided into urgent, time-critical and non-time critical data, in descending order of priority. Each time slot in the CFP is dedicated to a node that might transmit urgent data. To increase transmission opportunities, urgent data can be transmitted over the unused CFP time slots. Since the urgent data are sporadically generated, the slots of CFP can be further reserved for time-critical data (*i.e.*, the slots are dually reserved) in order to increase the overall throughput.

The simulation results show that our protocol is very effective and provides significantly shorter delay by providing opportunistic transmission chances. In the simulation, CoR-MAC shows an approximate 50%–850% better performance in the delay of urgent and time-critical data. Furthermore, our protocol provides flexible power consumption compared to the existing protocols.

## Figures and Tables

**Table 1:** Theoretical approach parameters.

Parameter	Value
*N*	Number of nodes
*L*	Number of slots in CAP
$P_{f}$	Probability of error or collision when the packet is transmitted
$P_{w}$	Probability of winning a contention
$\lambda_{u}$	Arrival rate of urgent data
$\lambda_{n}$	Arrival rate of non-time critical data
$T_{sf}$	Length of the superframe period
$T_{b}$	Length of the beacon period
$T_{i}$	Length of the inactive period
$T_{send}$	Transmission time in CAP
$\tau_{cfp}$	Length of a CFP slot
$\tau_{cap}$	Length of a system time slot

**Table 2:** Simulation parameters.

Parameter	Value
Topology Size	2 m × 0.5 m
Number of Sensor Node	1–20
Number of Coordinator	1
Arrival Rate of	2 frames /
Urgent Data	s per node
Arrival Rate of	20 frames /
Time-Critical Data	s per node
Arrival Rate of	200 frames /
Non-Time-Critical Data	s per node
Superframe Period ($T_{sf}$)	20 ms
Beacon Period ($T_{b}$)	450 μs
CFP Time Slot Length	843.9 μs
Link Data Rate	971.4 kbps
Beacon Frame Size	128–400 bits
RTS Frame Size	24 bits
CTS Frame Size	24 bits
Data Frame Size	192 bits
ACK Frame Size	24 bits
System time slot	5 μs
SIFS	20 μs
MIFS	75 μs
LIFS	150 μs
Power Supply	1.8 V
Power Consumption in Tx Mode	8.5 mA
Power Consumption in Rx Mode	7 mA
Power Consumption in	1 μA
Inactive Mode	

**Table 3:** Delay statistics of urgent data with 15 nodes.

	CoR-MAC	IEEE 802.15.4	IEEE 802.15.6
Average Delay	0.885 ms	28.449 ms	21.133 ms
Minimum Delay	0.272 ms	3.416 ms	3.168 ms
Maximum Delay	1.573 ms	37.453 ms	29.857 ms
Percentage over Deadline	0%	32.168%	13.167%
